# CO_2_ Hydrogenation to Methanol over Cd_4_/TiO_2_ Catalyst: Insight into Multifunctional Interface

**DOI:** 10.1002/cctc.202101646

**Published:** 2022-01-27

**Authors:** Guanna Li, Jittima Meeprasert, Jijie Wang, Can Li, Evgeny A. Pidko

**Affiliations:** ^1^ Biobased Chemistry and Technology Wageningen University & Research Bornse Weilanden 9 6708WG Wageningen The Netherlands; ^2^ Laboratory of Organic Chemistry Wageningen University & Research Stippeneng 4 6708WE Wageningen The Netherlands; ^3^ Inorganic Systems Engineering Department of Chemical Engineering Delft University of Technology Van der Maasweg 9 2629 HZ Delft The Netherlands; ^4^ State Key Laboratory of Catalysis Dalian Institute of Chemical Physics Chinese Academy of Sciences 457 Zhongshan Road Dalian 116023 P. R. China

**Keywords:** CO_2_, hydrogenation, CH_3_OH, Cd_4_/TiO_2_, multifunctional interface

## Abstract

Supported metal catalysts have shown to be efficient for CO_2_ conversion due to their multifunctionality and high stability. Herein, we have combined density functional theory calculations with microkinetic modeling to investigate the catalytic reaction mechanisms of CO_2_ hydrogenation to CH_3_OH over a recently reported catalyst of Cd_4_/TiO_2_. Calculations reveal that the metal‐oxide interface is the active center for CO_2_ hydrogenation and methanol formation via the formate pathway dominates over the reverse water‐gas shift (RWGS) pathway. Microkinetic modeling demonstrated that formate species on the surface of Cd_4_/TiO_2_ is the relevant intermediate for the production of CH_3_OH, and CH_2_O^#^ formation is the rate‐determining step. These findings demonstrate the crucial role of the Cd‐TiO_2_ interface for controlling the CO_2_ reduction reactivity and CH_3_OH selectivity.

## Graphical abstract

**Multifunctional catalysts**: The detailed reaction mechanism of CO_2_ hydrogenation to CH_3_OH was investigated over the multifunctional catalyst of Cd_4_/TiO_2_. Benefiting from the cooperation between Lewis acids from the Cd and Ti sites and Lewis bases from TiO_2_ surface oxygen atoms, the Cd_4_/TiO_2_ exhibited high activity and selectivity for the CH_3_OH production.

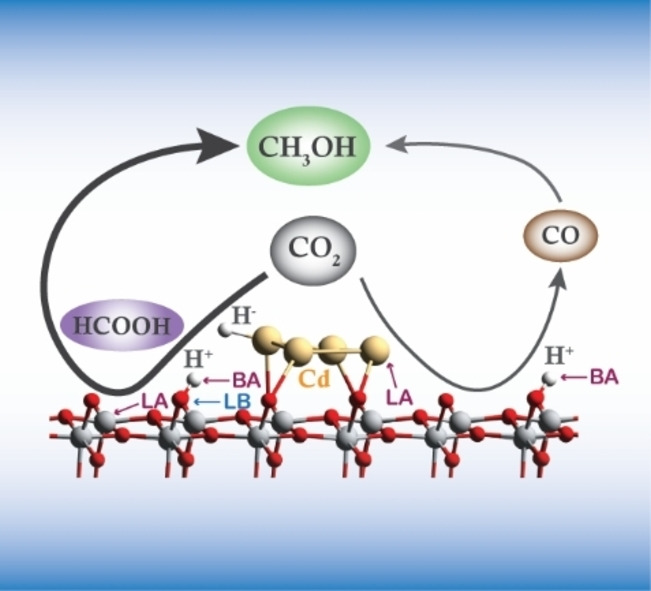

## Introduction

The increase of CO_2_ concentration in the atmosphere is one of the major factors in global climate change. CO_2_ capture and valorization have been considered as promising strategies to mitigate this problem.^[1]^ Using CO_2_ as a feedstock to produce valuable chemicals not only can help to decrease dramatically the amount of CO_2_ emitted into the atmosphere but also provide economic benefits.[^1b^, ^2^] A large number of value‐added chemicals can be produced from CO_2_ via platform molecules such as CO, CH_4_, and CH_3_OH.^[3]^ Among these, CH_3_OH is highly desirable because it is an important fuel as well as a starting feedstock for the production of more valuable chemical compounds.^[4]^ Recently, two different approaches for CO_2_ hydrogenation to CH_3_OH have received a lot of attention: (1) electrochemical reduction and (2) thermochemical reduction.^[5]^ The electrochemical CO_2_ reduction offers the advantage that product distribution can be controlled by adjusting electrolyte, electrocatalyst, and applied voltage.^[6]^ However, the selectivity, energetic efficiency, electrode lifetime restrict to its large‐scale applications.[^6c^, ^6d^] Therefore, using the thermochemical approach to synthesize CH_3_OH from CO_2_ hydrogenation is more practical for potential industrial applications compared to the alternative electrochemical CO_2_ reduction. It offers an opportunity for the development of sustainable technologies and environmentally benign chemical processes since H_2_ which is a reducing agent can readily be obtained from renewable energy resources.[^1a^, ^2^]

Many studies have been devoted to creating new tailor‐made CO_2_ conversion catalysts with improved activity and selectivity to methanol, of which Cu/ZnO/Al_2_O_3_ catalyst has been industrialized.^[7]^ However, the disadvantages of low CH_3_OH selectivity and the sintering of Cu and ZnO motivated the development of new Cu‐based catalysts such as Cu/ZnO,^[8]^ Cu/ZrO_2_,^[9]^ and Cu/CeO_2_.^[10]^ In these catalytic systems, it was found that H_2_ molecule is dissociated at the Cu site and CO_2_ is activated at the oxide surface, while the interface between Cu and metal oxide supports plays a crucial role for stabilization of the reaction intermediate for CH_3_OH formation.^[9]^ Besides Cu‐based catalysts, various other materials have also been reported as promising catalysts for CO_2_ hydrogenation to CH_3_OH. For instance, Au,^[11]^ Pd^[12]^, Re,^[13]^ ZnO^[14]^ and In_2_O_3_ ^[15]^ supported on oxides were reported to be active toward the production of CH_3_OH under moderate conditions. Although many different types of catalysts have been reported, all of the active sites involved in the reaction have a common feature of multi‐functionality in nature. An efficient cooperation between active sites of different catalytic natures coupled in one heterogeneous catalyst plays a key role for eventual selective CH_3_OH formation.

Regarding the reaction mechanism, typically, two different reaction pathways have been proposed for the hydrogenation of CO_2_ to CH_3_OH: (1) the reverse water‐gas shift (RWGS) pathway, and (2) the formate pathway. In the RWGS reaction, CO_2_ is hydrogenated to form CO* intermediate which is then further hydrogenated to form CH_3_OH. For the formate pathway, CH_3_OH is produced via the formate (HCOO*) intermediate.^[16]^ Most studies have suggested that the formate pathway is preferred over the RWGS pathway.[^7b^, ^12a^, ^17^] The main reason is that the binding strength of CO* intermediate on these catalysts is quite weak, leading to the desorption of CO to the gas phase. However, on some other catalysts such as Cu/TiO_2_, Cu/ZrO_2_, and Cu/CeO_x_, CH_3_OH was produced through CO* intermediate due to the strong enough interaction between CO* and catalyst.[^9b^, ^10^] Therefore, the specific reaction pathway dominating methanol formation is system‐dependent and should be investigated individually.

Recently we have investigated CO_2_ conversion to CH_3_OH on Cd/TiO_2_ and CdTiO_3_ catalysts by a combination of experimental and computational studies.^[18]^ It was found that Cd/TiO_2_ catalyst exhibits a much higher catalytic CO_2_ hydrogenation activity than the CdTiO_3_ mixed oxide. To further identify the detailed reaction mechanism catalyzed by Cd/TiO_2_ and clarify the functionalities of different types of active sites in this system, we constructed a Cd/TiO_2_ model catalyst and investigated its catalytic activity towards CO_2_ conversion to methanol with H_2_ as a reductant. The key objective of this study is to explore the multiple‐site cooperation effects on the catalyst reactivity by combining DFT calculation with microkinetic modeling.

## Results and Discussion

### Cd_4_/TiO_2_ Model rationalization

**Figure 1:**
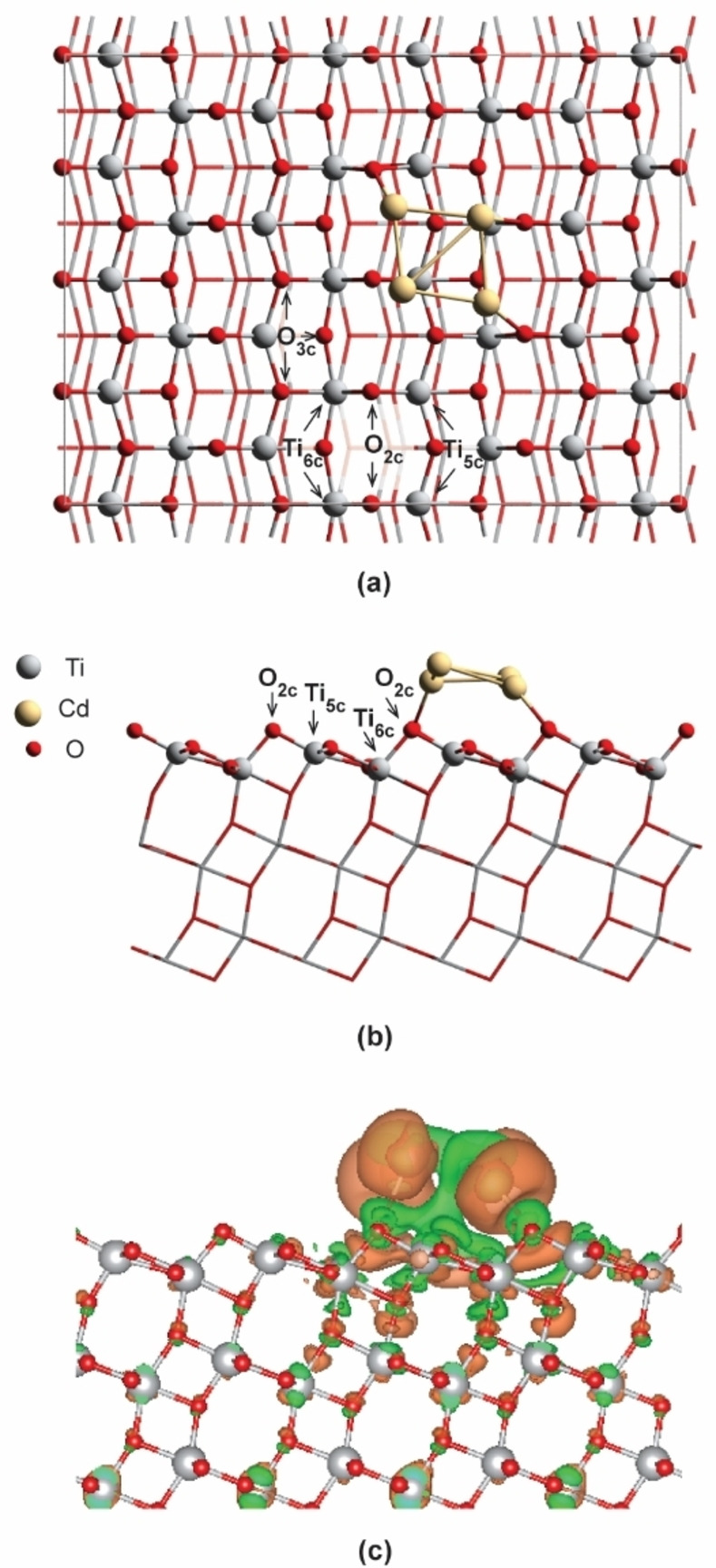
**(a)** Top view and **(b)** side view of Cd_4_/TiO_2_(101) slab model. The O_2c_ and O_3c_ are twofold coordinated and threefold coordinated oxygen atoms, and the Ti_5c_ and Ti_6c_ are fivefold coordinated and sixfold coordinated titanium atoms on the surface of TiO_2_, respectively. **(c)** The electron density different plots upon the adsorption of Cd_4_ cluster on TiO_2_ surface. The orange and green regions represent electrons depletion and accumulation respectively (isosurface value=0.05 e/Bohr^3^).

A cluster containing 4 Cd atoms (Cd_4_) was selected as representative of the supported Cd nanoparticles on the TiO_2_ surface since it was reported as the smallest Cd cluster featuring a magic number of Cd atoms. In order to model the Cd_4_/TiO_2_ catalyst, two possible configurations of isolated Cd_4_ cluster, i. e., a tetrahedron (T_d_) and planar rhombus (C_2V_)^[28]^ were firstly optimized in the vacuum by using a large unit cell of 15×15×15 Å (Figure S1 in the supporting information). Then the so‐obtained Cd_4_ clusters were deposited and optimized on the (101) surface of anatase TiO_2_. It is found that the most stable configuration of the supported Cd_4_ cluster on the (101) TiO_2_ surface is a deformed planar geometry even though the tetrahedron is more stable in the gas phase. As shown in Figure 1, the Cd_4_ (C_2V_) cluster is slightly distorted upon the adsorption with one of the Cd atoms lying above the plane of the other three. The adsorption energy of Cd_4_ over the surface is calculated to be −1.05 eV indicating a strong interaction between the metal cluster and the support of TiO_2_. Bader charge analysis demonstrates that the entire Cd_4_ cluster is +1.48|e| charged, which indicates that the electrons are transferred from Cd_4_ cluster to TiO_2_ surface through metal‐support interaction.

### H_2_ dissociation and H spillover

**Figure 2:**
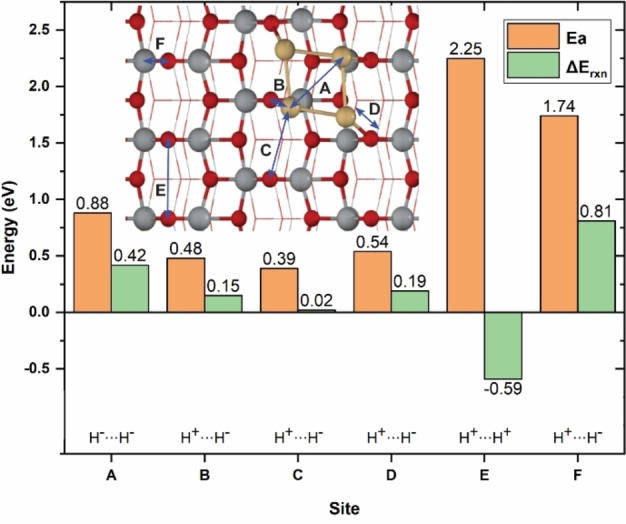
Activation energy (Ea) and reaction energy (ΔE_rxn_) for H_2_ dissociation at all possible active sites of Cd_4_/TiO_2_ catalyst.

Many studies have proposed that activation and dissociation of an H_2_ molecule take place at the metal‐oxide interface.^[29]^ In this work, six possible active sites of Cd_4_/TiO_2_ catalyst for the activation and dissociation of H_2_ molecule were systematically studied. As shown in Figure 2, site A is on top of the supported Cd_4_ cluster. Site B, C and D are at the interface of Cd_4_/TiO_2_ (Cd−O_2c_). Site E is located between two nearest O_2c_ atoms and site F is on top of bridging Ti_5c_ and O_2c_ atoms of TiO_2_ surface. From Figure 2 it can be seen that that heterolytic H_2_ dissociation at the interface of Cd/TiO_2_ is more preferable than the other sites. Among all interface sites considered, H_2_ dissociation over site C has the lowest activation barrier (0.39 eV). Homolytic dissociation of H_2_ molecule over site A needs to overcome an activation barrier of 0.88 eV and generates two hydrides on the supported Cd_4_ cluster. On the TiO_2_ surface, both homolytic (site E) and heterolytic dissociation pathways (site F) exhibit very high activation barriers (1.74 and 2.25 eV) indicating that TiO_2_ surface site is inactive for H_2_ activation. This is in agreement with a previous theoretical study of H_2_ dissociation on TiO_2_ surfaces.^[30]^

After we figured out the most favorable active site for H_2_ dissociation, the spillover process of the so‐formed H* on the surface of the Cd_4_/TiO_2_ is further studied. As shown in Figure S2, the migration of the H* generated by H_2_ dissociation at the interface (site C) from O_2c_ site to its neighboring O_3c_ site has an activation barrier of 0.73 eV. The other H* species on the Cd_4_ cluster can also spillover to the surface of TiO_2_ with an activation barrier of 0.71 eV. H* on O_3c_ site can also hoop to another O_2c_ site next to it by overcoming a barrier of only 0.42 eV. The overall reaction is slightly endothermic. These results indicate that the activated H* on the surface of the Cd_4_/TiO_2_ catalyst is rather dynamic and hydrogen migrations among different surface sites is thermodynamically and kinetically easy processes.

### CO_2_ hydrogenation to HCOOH and CO

In this section, the hydrogenation of CO_2_ on Cd_4_/TiO_2_ catalyst will be discussed. Two main reaction pathways of CO_2_ hydrogenation which have been intensively debated in the literature were studied: (1) methanol formation via the intermediate of HCOOH*, and (2) methanol formation via the reversed water‐gas shift (RWGS) pathway with CO as an intermediate. Noted that the species with asterisk (*) and hash sign (^#^) are species that interact with TiO_2_ surface and Cd_4_ cluster of the Cd_4_/TiO_2_ catalyst, respectively.

#### Formate pathway

**Figure 3:**
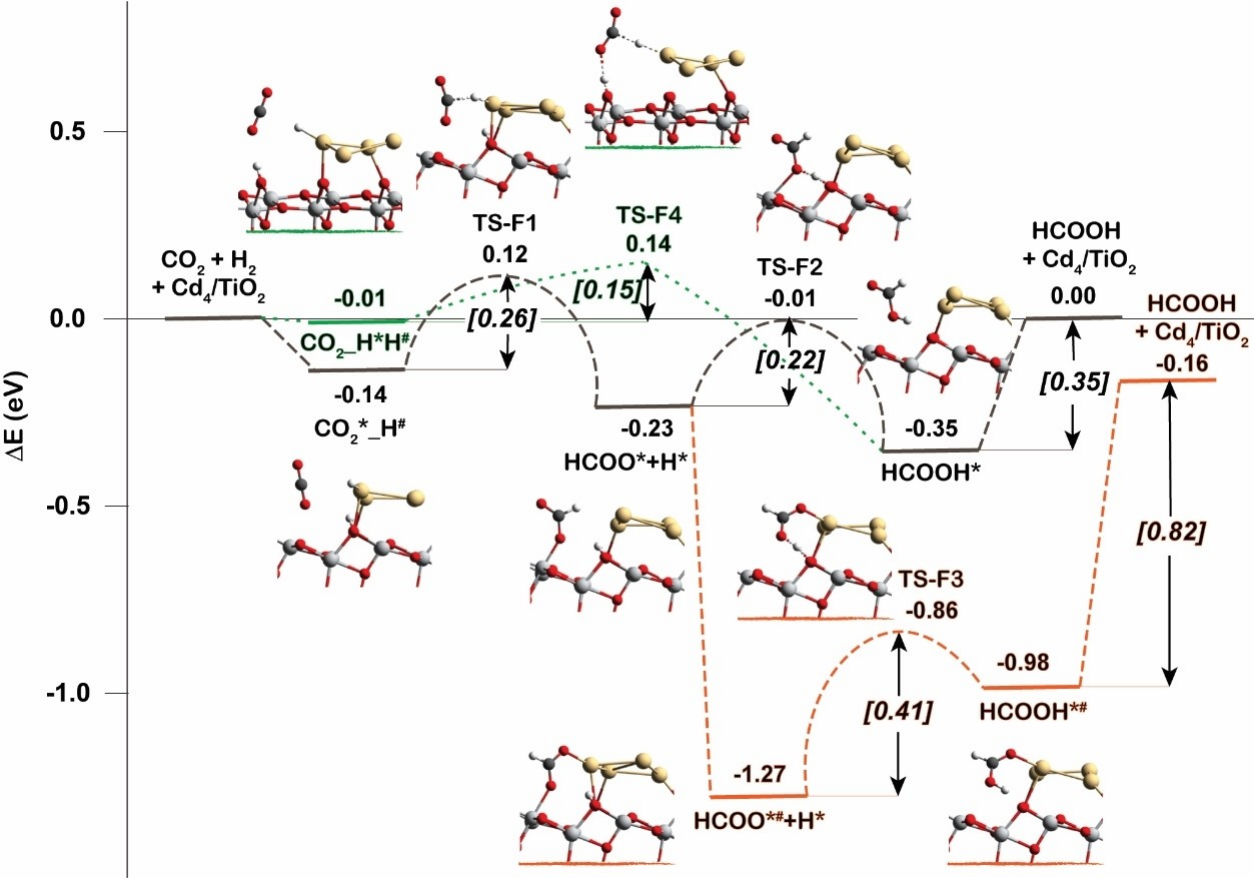
Reaction energy profiles for the CO_2_ hydrogenation to HCOOH* on Cd_4_/TiO_2_ catalyst. Green line is the concerted pathway. Gray line is the stepwise pathway via monodentate HCOO*. Orange line is the stepwise pathway via bidentate HCOO*^#^. The species with asterisk (*) and hash sign (^#^) are species that interact with TiO_2_ surface and Cd_4_ cluster of the Cd_4_/TiO_2_ catalyst, respectively.

The reaction mechanisms of CO_2_ hydrogenation to formate (HCOO*) and formic acid (HCOOH*) are shown in Figure 3. After heterolytic dissociation of H_2_ at the interface of Cd_4_/TiO_2_, a hydride coordinated to Cd (H^#^) and a proton bonded to O_2c_ site (H*) are produced. CO_2_ is adsorbed over the Ti_5c_ site nearby both H* and H^#^ species. The adsorption energy is calculated to be −0.14 eV. Then CO_2_ can be hydrogenated by the transfer of H^#^ from Cd_4_ cluster to the C atom of CO_2_ forming formate intermediate of HCOO*. The activation barrier for this step is only 0.26 eV. Further protonation of HCOO* to form formic acid (HCOOH*) can be realized via two different reaction routes, either by protonation of monodentate HCOO* intermediate to form *cis*‐HCOOH* (gray line in Figure 3), or protonation of bidentate HCOO*^#^ intermediate which can be formed by structure rearrangement to form *trans*‐HCOOH*^#^ (orange line in Figure 3). The activation barriers of proton transfer for both routes are relatively low (0.22 and 0.41 eV), however, the configurational transformation of HCOO* from monodentate coordination to bidentate coordination with both Ti_5c_ and Cd before protonation reaction is dramatically favorable. Another possible pathway for HCOOH* formation is also identified with a small activation barrier of 0.15 eV, the so‐called concerted reaction mechanism with CO_2_ hydrogenation by both H* and H^#^ in one step (green line in Figure 3).

#### RWGS pathway

**Figure 4:**
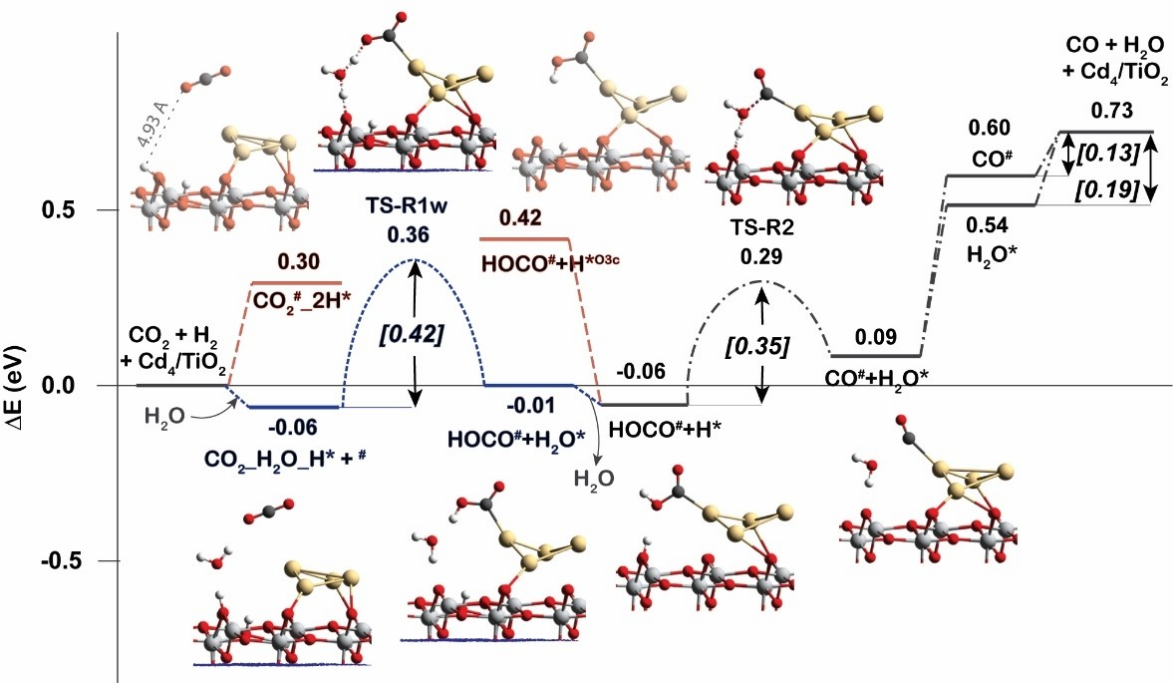
Reaction energy profiles for the CO_2_ hydrogenation to CO on Cd_4_/TiO_2_ catalyst (RWGS pathway). Blue line is the reaction with the assist of H_2_O molecule.

The RWGS reaction mechanism is initiate by CO_2_ hydrogenation to first form carboxylate intermediate (HOCO^#^), from which CO is produced and can be further converted into methanol by continuous hydrogenation reactions. As shown in Figure 4, the reaction starts with the adsorption of CO_2_ at the perimeter site of Cd_4_ cluster after hydrogen spillover process. Then, the CO_2_ can be protonated by the H* on TiO_2_ surface forming HOCO^#^. It is found that this reaction cannot occur directly due to the long distance between CO_2_ and H* (4.93 Å). However, it can proceed by the assist of an H_2_O molecule which acts as a proton shuttle between H* and CO_2_ (blue line in Figure 4). The activation energy in this case is calculated to be 0.42 eV indicating that this process is feasible. Subsequent hydrogenation of the HOCO^#^ intermediate at its terminal OH group with the breaking of C−O bond produces CO^#^ and H_2_O*. This process requires overcome an activation barrier of 0.35 eV. Finally, CO and H_2_O can be desorbed from the catalyst with desorption barriers of 0.13 and 0.19 eV, respectively.

**Figure 5:**
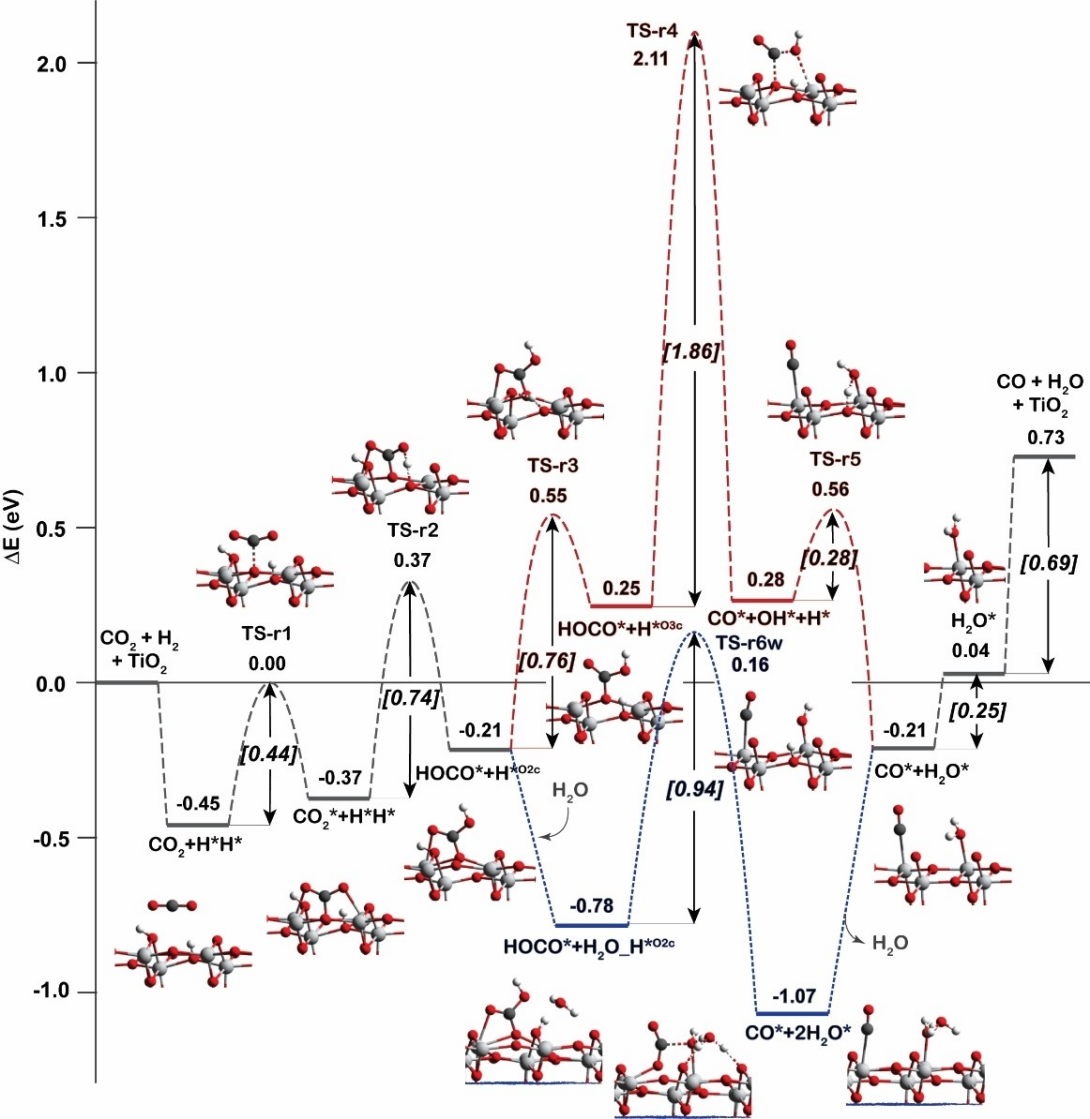
Reaction energy profiles for the CO_2_ hydrogenation to CO on clean TiO_2_ surface (RWGS pathway). Blue line is the reaction with the assist of H_2_O molecule.

Due to the unfavorable adsorption of CO_2_ on the supported Cd_4_ cluster, we also explored the CO_2_ adsorption on a separate TiO_2_ surface site without interaction with the Cd_4_ cluster. The mechanisms of RWGS reaction on the TiO_2_ surface are shown in Figure 5. In this case this reaction starts with the adsorption of CO_2_ on the TiO_2_ surface after hydrogen spillover process. The adsorption energy of CO_2_ is calculated to be −0.45 eV which is relatively stronger than that on supported Cd_4_ cluster. The bent CO_2_ geometry can be formed on the TiO_2_ surface with an activation barrier of 0.44 eV. Then the adsorbed CO_2_* is directly hydrogenated to form HOCO* without the H_2_O mediator. The activation energy for this step is calculated to be 0.74 eV. The diffusion of the second H* to the O_3c_ site close to the OH group of HOCO* needs overcome an activation barrier of 0.76 eV. After that, the cleavage of the C−O bond of HOCO* intermediate to generate CO* and OH* species on the TiO_2_ surface is rather difficult with an activation barrier of 1.86 eV. However, the presence of H_2_O molecule can again decrease this activation barrier to 0.94 eV with C−O bonding breaking and OH group hydrogenation occurring simultaneously.

It is found that H_2_O molecule plays an important role as a proton shuttle to promote the most difficult reaction steps during the RWGS reactions taking place at both interface and TiO_2_ surface of Cd_4_/TiO_2_ catalyst. The hydrogenation reaction of CO_2_ is the most difficult step for the reaction occurred at the interface while the C−O bond cleavage of HOCO* carboxylate intermediate is found to be the most difficult step for the reaction occurred at the TiO_2_ surface. The highest activation energy of the RWGS reaction that occurs at the interface of Cd_4_ and TiO_2_ surface (TS‐R1w) is about two times lower than that of the other reaction route on the TiO_2_ surface (TS‐r6w). Therefore, it is concluded that the most preferable active site for the RWGS reaction is the interface of Cd_4_/TiO_2_ catalyst. Therefore, in the next section, the discussion of CH_3_OH formation via CO* will only focus on the reaction route at the interface.

### CH_3_OH formation

**Figure 6:**
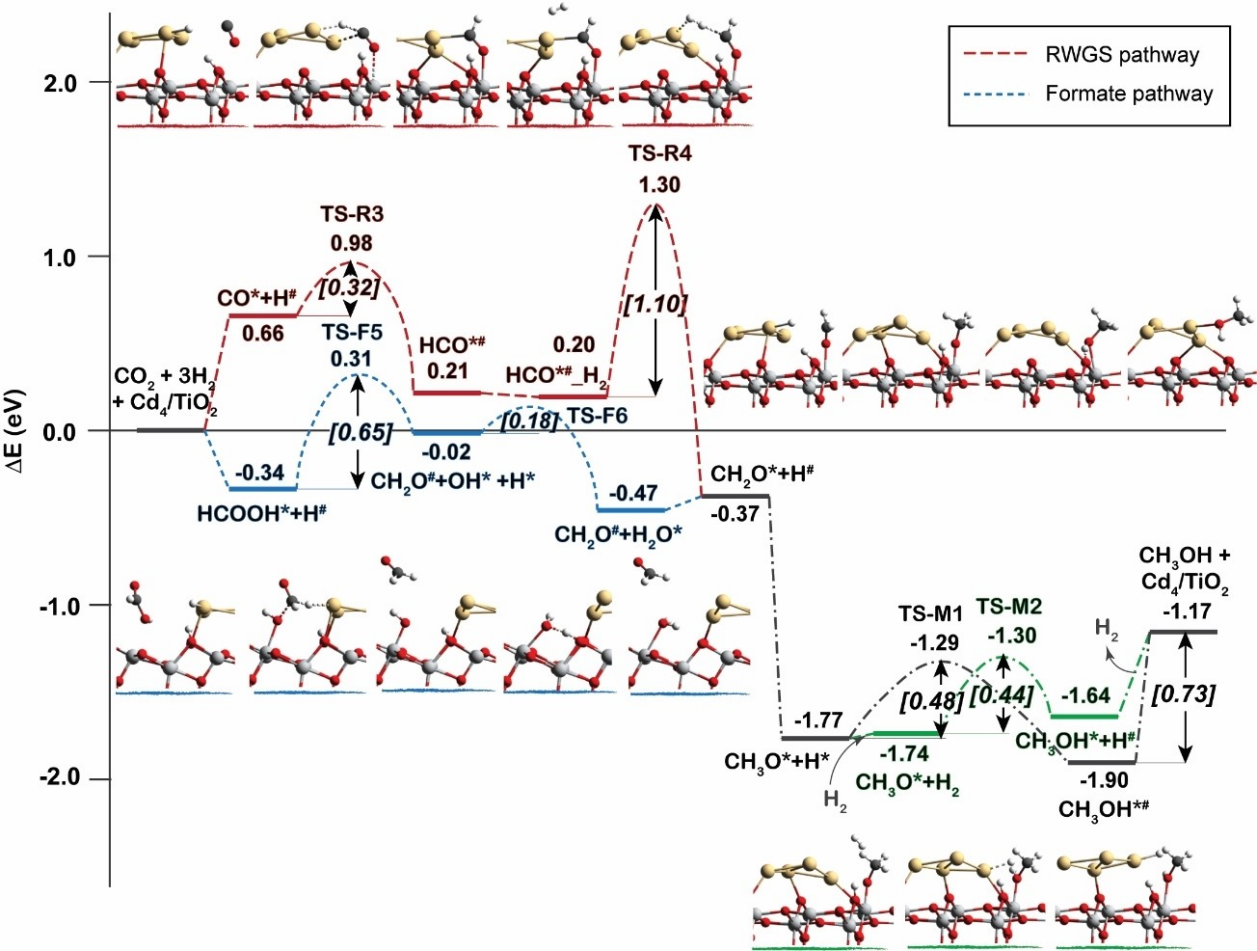
Reaction energy profiles for the production of CH_3_OH from CO and HCOOH.

In this section, we will discuss the reaction mechanism of CH_3_OH formation from HCOOH* as well as CO* intermediates generated from the formate and the RWGS reaction pathways. The results are shown in Figure 6. Totally 4 elementary hydrogenation reaction steps are involved for CH_3_OH formation from CO i. e. CO*→HCO*^#^, HCO*^#^→CH_2_O*, CH_2_O*→CH_3_O* and CH_3_O*→CH_3_OH. The activation barrier for CO hydrogenation to form HCO*^#^ is 0.32 eV by H^#^ on Cd_4_ cluster. The next step of dissociative adsorption of H_2_ on top of HCO*^#^ intermediate generating CH_2_O* and H^#^ species has an activation barrier of 1.10 eV. Subsequent CH_3_O* formation by CH_2_O* hydrogenation is a barrierless process with a reaction energy of −1.40 eV. Finally, the CH_3_OH is formed by hydrogenation of CH_3_O* intermediate with the activation barrier of 0.48 eV. In addition, CH_3_OH can be produced by the hydrogenolysis of CH_3_O* (green line in Figure 6). The activation energy of this step is only 0.04 eV lower than that of the CH_3_O* hydrogenation step. These results imply that both CH_3_O* hydrogenolysis and CH_3_O* hydrogenation coexist in the formation of CH_3_OH.

Alternatively, CH_3_OH can also be formed from HCOOH* (blue line in Figure 6). The initial step is the hydrogenation of HCOOH* to produce formaldehyde (CH_2_O^#^) and an OH* species (CH_2_O^#^+OH*+H*). The activation energy of this step is calculated to be 0.65 eV. Then, the OH* is protonated to form H_2_O and regenerate a vacant interfacial active site on the surface. In the next step, after another H_2_ molecule is dissociated at the interface, the CH_3_OH can be formed by two successive hydrogenation steps from CH_2_O*, which is the same process as the reactions via the RWGS pathway.

**Figure 7:**
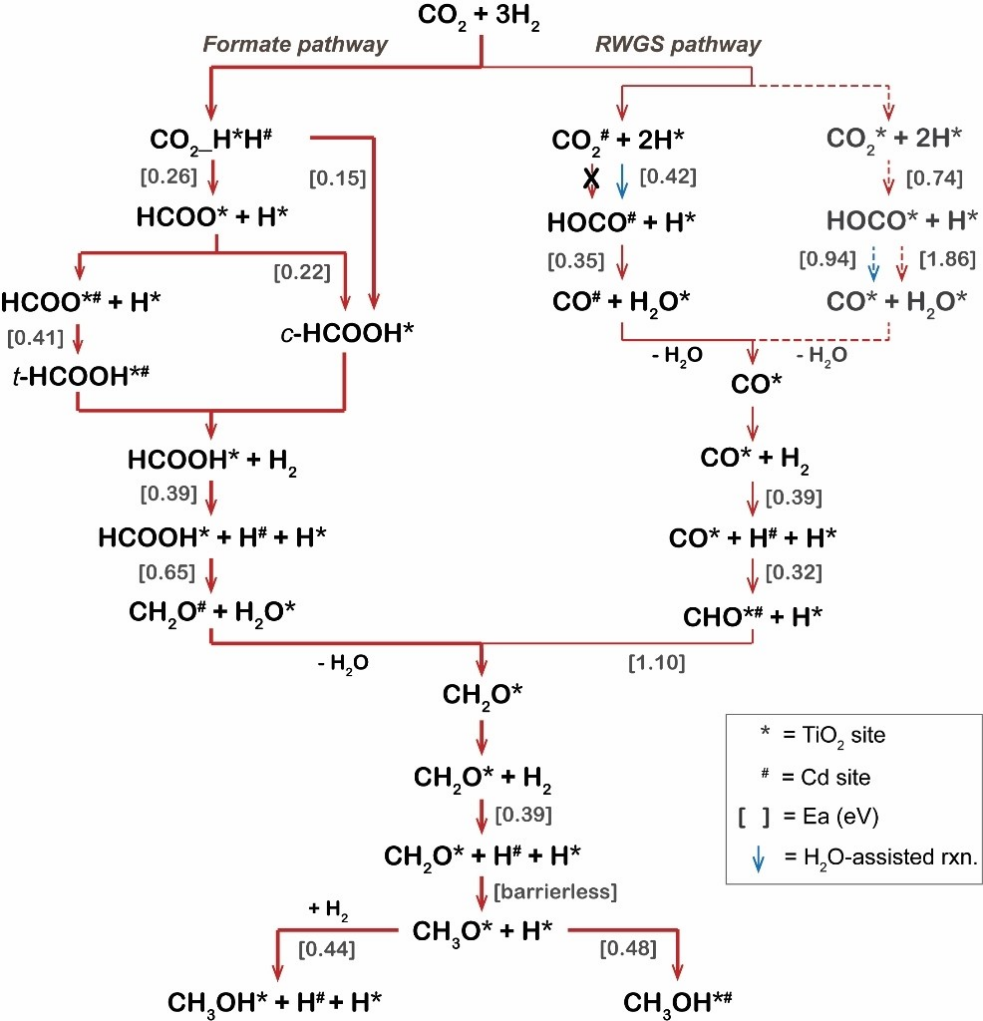
A schematically representation of the whole reaction mechanism for CO_2_ hydrogenation to CH_3_OH on Cd_4_/TiO_2_ catalyst. Numbers in parenthesis represent activation energies in eV. Solid lines and dash lines represent reaction that occurs at the interface and TiO_2_ surface, respectively.

To summarize, Figure 7 gives a schematical representation of the whole DFT reaction mechanism identified in this work, and the whole reaction pathways of CO_2_ hydrogenation to CH_3_OH on Cd_4_/TiO_2_ catalyst is shown in Figure S4. It can be seen that the formate pathway dominates over the RWGS pathway for the production of CH_3_OH from CO_2_ and H_2_. The formation of CH_2_O* intermediate is found to be the most difficult reaction step for CH_3_OH production from both RWGS and formate reaction routes.

### Microkinetic modeling

**Figure 8:**
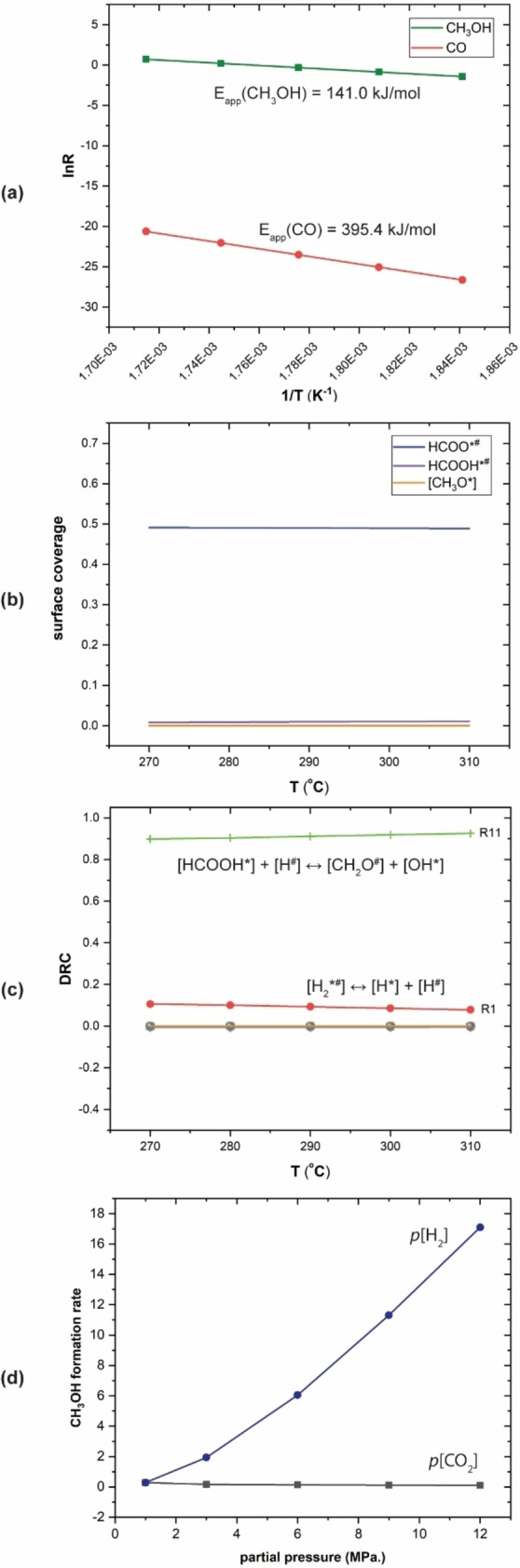
Results of the microkinetic modeling for the CO_2_ hydrogenation on Cd/TiO_2_ catalyst. **(a)** is product formation rates as a function of temperature (T=270–310 °C) and the calculated apparent activation energy (E_app_). **(b)** is surface coverages of major surface intermediates at 270–310 °C. **(c)** is degree of rate control analysis at 270–310 °C. **(d)** is the partial pressure dependence of the CH_3_OH formation rate at 290 °C. The partial pressure of another reactant is fixed as 1 MPa.

All considered elementary steps of the CO_2_ hydrogenation to CH_3_OH on Cd_4_/TiO_2_ catalyst, and corresponding activation energies are listed in Table 1. The MKM is performed using a dual‐site model representing TiO_2_ (*) and Cd (^#^) sites on Cd_4_/TiO_2_ catalyst, respectively. The ratio between the number of * and ^#^ sites is 0.5 : 0.5. The reaction rate, surface coverages, and degree of rate control (DRC) are calculated under the following steady‐state reaction conditions: total pressure=2 MPa., H_2_/CO_2_=3 : 1, temperature=270–310 °C. The apparent activation energy (E_app_) is determined from the slope of the Arrhenius plot, as shown in Figure 8a. The E_app_ for the CH_3_OH formation is calculated to be 1.46 eV (141.0 kJ/mol), while that for the CO formation is much higher, 4.10 eV (395.4 kJ/mol). This agrees with the experiment that the Cd/TiO_2_ catalyst exhibits high CH_3_OH selectivity (70 %).^[18]^ These results also indicate that the reaction rate of products increases with the increasing of reaction temperature.

**Table 1:** Summary of elementary reaction steps and activation energies from DFT calculations used for microkinetic modeling. Ea–f and Ea–b are activation energy for forward and backward reaction, respectively. * and ^#^ represent TiO_2_ and Cd sites on Cd_4_/TiO_2_ catalyst.

	Elementary reaction step	Ea–f [eV]	Ea–b [eV]
	H_2_ dissociation		
R0:	[H_2_]+[*]+[^#^]↔[H_2_*^#^]	0.00	0.02
R1:	[H_2_*^#^]↔[H*]+[H^#^]	0.39	0.38
R2:	[H^#^]+[*]↔[H*]+[^#^]	0.71	0.78
	Formate pathway 1 *(CO_2_ to HCOOH)*		
R3:	[CO_2_]+[H*]+[H^#^]↔[CO_2__H*H^#^]	0.00	0.01
R4:	[CO_2__H*H^#^]↔[HCOOH*]+[^#^]	0.15	0.49
R5:	[CO_2_]+[*]+[H^#^]↔[CO_2_*_H^#^]	0.00	0.14
R6:	[CO_2_*_H^#^]↔[HCOO*]+[^#^]	0.26	0.35
R7:	[HCOO*]+[H*]↔[HCOOH*]+[*]	0.22	0.34
R8:	[HCOO*]+[^#^]↔[HCOO*^#^]	0.00	1.04
R9:	[HCOO*^#^]+[H*]↔[HCOOH*^#^]+[*]	0.41	0.12
R10:	[HCOOH*^#^]↔[HCOOH*]+[^#^]	0.63	0.00
	Formate pathway 2 *(HCOOH to CH_2_O)*		
R11:	[HCOOH*]+[H^#^]↔[CH_2_O^#^]+[OH*]	0.65	0.33
R12:	[CH_2_O^#^]+[*]↔[CH_2_O*]+[^#^]	0.00	0.44
R13:	[OH*]+[H*]↔[H_2_O*]+[*]	0.18	0.63
R14:	[H_2_O]+[*]↔[H_2_O*]	0.00	0.57
	CH_3_OH formation *(CH_2_O to CH_3_OH)*		
R15:	[CH_2_O*]+[H^#^]↔[CH_3_O*]+[^#^]	0.00	1.40
R16:	[CH_3_O*]+[H*]+[^#^]↔[CH_3_OH*^#^]+[*]	0.48	0.61
R17:	[CH_3_OH]+[*]+[^#^]↔[CH_3_OH*^#^]	0.00	0.73
	RWGS pathway 1 *(CO_2_ to CO)*		
R18:	[H_2_O]+[H*]↔[H_2_O_H*]	0.00	0.26
R19:	[CO_2_]+[H_2_O_H*]↔[CO_2__H_2_O_H*]	0.00	0.06
R20:	[CO_2__H_2_O_H*]+[^#^]↔[HOCO^#^]+[HOH*]	0.42	0.37
R21:	[H_2_O]+[*]↔[HOH*]	0.00	0.19
R22:	[HOCO^#^]+[H*]↔[CO^#^]+[HOH*]	0.35	0.20
R23:	[CO]+[^#^]↔[CO^#^]	0.00	0.13
	RWGS pathway 2 *(CO to CH_2_O)*		
R24:	[CO]+[*]↔[CO*]	0.00	0.03
R25:	[CO*]+[H^#^]↔[HCO*^#^]	0.32	0.77
R26:	[HCO*^#^]+[H_2_]↔[HCO*^#^_H_2_]	0.00	0.01
R27:	[HCO*^#^_H_2_]↔[CH_2_O*]+[H^#^]	1.10	1.67
	CH_3_O* hydrogenolysis to CH_3_OH		
R28:	[CH_3_O*]+[H_2_]+[^#^]↔[CH_3_OH*]+[H^#^]	0.44	0.34
R29:	[CH_3_OH]+[*]↔[CH_3_OH*]	0.00	0.46

Figure 8b shows that the HCOO*^#^ has the highest surface coverage (σ≈0.5), indicating the formation of this intermediate is the resting state of the overall reaction. This results is consistent with the experimental in‐situ IR observation.^[18]^ DRC analysis (Figure 8c) shows that the conversion of HCOOH* to CH_2_O^#^ (R11), the most difficult reaction step of the formate pathway, is also the rate‐determining step. This result demonstrates that formate pathway dominates over RWGS pathway for the CO_2_ hydrogenation to CH_3_OH on the surface of Cd/TiO_2_ catalyst. In addition, it was found that the H_2_ dissociation reaction step (R1 in Table 1) has only a minor influence on the overall reaction rate. The effect of H_2_ and CO_2_ partial pressure on the reaction rate is also investigated by MKM, as shown in Figure 8d. These results indicate that increasing H_2_ partial pressure can enhance significantly the methanol production rate, which, in turn, is not affected by the CO_2_ partial pressure.

## Conclusion

In conclusion, the reaction mechanisms of CO_2_ hydrogenation to methanol by H_2_ have been investigated in this study by comprehensive DFT and MKM. It is proposed that the interface between the Cd_4_ cluster and the support of TiO_2_ plays a key role for H_2_ dissociation as well as preactivation of CO_2_. H_2_ dissociation and CO_2_ activation are energetically more favorable at the Cd‐TiO_2_ interface than that at bare TiO_2_ surface and Cd cluster. Both CO_2_ hydrogenation reactions to formate and CO are remarkably facilitated by the synergy between H^−^ on Cd and H^+^ on TiO_2_ surface (Figure 3, formate pathway; Figure 4, RWGS pathway). In contrast, CO_2_ conversion to CH_3_OH on bare TiO_2_ is very difficult compared to the Cd/TiO_2_ interface. Cd‐TiO_2_ interface is crucial for stabilizing various reaction intermediates and promoting the rate‐determining step of formaldehyde formation identified by DFT and MKM. All these mechanism results indicates that the multifunctionality of Cd/TiO_2_ interface including Lewis acids of metals and Lewis base of surface oxygen is of great importance accounting for the outstanding catalytic activity of Cd/TiO_2_ material. Water molecules produced from the reaction or present in the reaction system can dramatically facilitate the most difficult reaction steps of RWGS reaction. However, formate is identified to be the relevant intermediate for CO_2_ hydrogenation to methanol, with formaldehyde formation being the rate‐limiting reaction step. Our results demonstrate that Cd/TiO_2_ can be a promising candidate for valorization of CO_2_ to produce methanol and the multifunctionality of the metal‐support interface is a crucial aspect for rational design of CO_2_ hydrogenation catalyst.

## Experimental Section

All DFT calculations have been performed using the Vienna Ab Initio Simulation Package (VASP).^[19]^ The generalized gradient approximation (GGA) with PBE exchange and correlation functional was used to account for the exchange‐correlation energy.[^19b^, ^20^] The kinetic energy cutoff of the plane wave basis set was set to 400 eV. The threshold for energy convergence for each iteration was set to 10^−5^ eV. Geometries were assumed to be converged when forces on each atom were less than 0.05 eV/Å. Gaussian smearing of the population of partial occupancies with a width of 0.10 eV was used during iterative diagonalization of the Kohn‐Sham Hamiltonian. The bulky TiO_2_ unit cell in the phase of anatase was firstly fully optimized. The optimized lattice vectors of a=3.799 Å b=3.799 Å c=9.716 Å have a good agreement with the experiment parameters.^[21]^ For Cd_4_/TiO_2_ model, 1x3 and 2×4 supercells of anatase TiO_2_ (101) surface with a vacuum space of 15 Å were built for investigation of the reaction mechanism of H_2_ dissociation and CO_2_ hydrogenation, respectively. These slab models contain six titanium layers with the bottom three layers were fixed while the rest was allowed to relax during the geometry optimization. The lattice parameters were fixed throughout the surface calculations. The nudged‐elastic band method with the improved tangent estimate (CI‐NEB) was used to determine the minimum energy path and to locate the transition state structure for each elementary reaction step.^[22]^ The maximum energy geometry along the reaction path generated by the NEB method was further optimized using a quasi‐Newton algorithm. In this procedure, only the extra‐framework atoms were relaxed. Vibrational frequencies were calculated by determining the second derivatives of the Hessian matrix using the density functional perturbation theory as implemented in VASP 5.3.5. Transition state was confirmed by showing a single imaginary frequency corresponding to each reaction coordinate. Bader charge analysis was visualized by VESTA software.^[23]^

Mean‐field microkinetic modeling (MKM) is applied based on the DFT calculations of all elementary reaction steps. The rate constant of the adsorption reaction is calculated by the Hertz‐Knudsen equation [Eq. 1]:^[24]^

(1) $$k_{ads}=\frac{PA}{\sqrt{2\pi mk_{b}T}}S$$

where $k_{ads}$ is the rate constant of adsorption reaction, P is the partial pressure of the adsorbate in the gas phase, A is the surface area of the adsorption site, m is the mass of adsorbate, $k_{b}$ is the Boltzmann constant, T is the temperature, and S is the sticking coefficient.

The desorption reaction is calculated by Equation 2:

(2) $$k_{des}=\frac{k_{b}T^{3}}{h^{3}}\frac{A\left(2\pi mk_{b}\right)}{\sigma \theta_{rot}}e^{\left(\frac{-E_{des}}{k_{b}T}\right)}$$

whare $k_{des}$ is the rate constant of desorption reaction, h is the Plank's constant, σ is the symmetry number of a molecule, $\theta_{rot}$ is the rotational temperature of a molecule, and $E_{des}$ is the desorption energy.

For the surface reaction, it is calculated by the Eyring equation [Eq. 3]:^[25]^

(3) $$k=\frac{k_{b}T}{h}e^{-\frac{E_{a}}{RT}}$$

where k is the rate constant of surface reaction, $E_{a}$ is the activation energy, and R is the gas constant.

The approach to MKM has been presented in detail elsewhere.^[26]^ The differential equations are constructed using the rate constants and the set of elementary reaction steps. For each of the M components in the kinetic network, a single differential equation is in the form [Eq. 4]:

(4) $$r_{i}=\sum_{j=1}^{N}\left(k_{j}v_{i}^{j}\prod_{k=1}^{M}c_{k}^{v_{k}^{j}}\right)$$

where $r_{i}$ is the rate reaction, $k_{j}$ is the elementary reaction constant, $v_{i}^{j}$ is the stoichiometric coefficient of component i in elementary reaction step k and $c_{k}$ is the concentration of component k on the catalytic surface.

The degree of rate control (DRC) was performed to investigate the elementary steps that contribute to the rate control over the overall reaction ref: [21–23[.^[27]^ For elementary step i , the degree of rate control $X_{RC,i}$ is defined as [Eq. 5]

(5) $$X_{RC,i}=\frac{k_{i}}{r}{\left(\frac{\partial r}{\partial k_{i}}\right)}_{k_{j\neq i,K_{i}}}={\left(\frac{\partial lnr}{\partial lnk_{i}}\right)}_{k_{j\neq i,K_{i}}}$$

where $k_{i},\;K_{i}$ and r are the rate constants, the equilibrium constant for step i and the reaction rate, respectively. All MKM results are simulated by a homemade script.

## Conflict of interest

The authors declare no conflict of interest.

1

## Supporting information

As a service to our authors and readers, this journal provides supporting information supplied by the authors. Such materials are peer reviewed and may be re‐organized for online delivery, but are not copy‐edited or typeset. Technical support issues arising from supporting information (other than missing files) should be addressed to the authors.

Supporting Information

## Data Availability

The data that support the findings of this study are available in the supplementary material of this article.
