# Supplementary material for: CO2 Hydrogenation to Methanol over Cd4/TiO2 Catalyst: Insight into Multifunctional Interface
Source: ChemCatChem. 2022 Jan 27;14(5):e202101646. doi: 10.1002/cctc.202101646 (PMC9305886; doi:10.1002/cctc.202101646)
Supplement: Supplementary file 1 — Supporting Information [file CCTC-14-0-s001.pdf]

ChemCatChem

Supporting Information

CO_2 Hydrogenation to Methanol over Cd_4/TiO_2 Catalyst: Insight into Multifunctional Interface

Guanna Li,* Jittima Meeprasert, Jijie Wang, Can Li, and Evgeny A. Pidko*

Author Contributions

J.M. Data curation:Lead; Formal analysis:Lead; Investigation:Lead; Visualization:Lead; Writing – original draft:Lead
J.W. Conceptualization:Equal; Supervision:Supporting; Writing – review & editing:Supporting
C.L. Conceptualization:Lead; Supervision:Supporting; Writing – review & editing:Supporting
E.P. Conceptualization:Lead; Supervision:Lead; Writing – review & editing:Lead

1. Cd_4 clusters

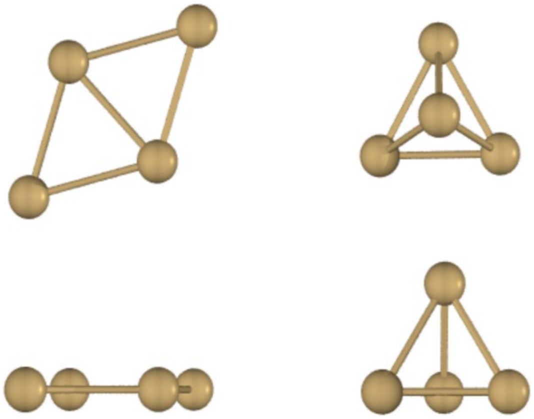

(a) (b)

Figure S1. The optimized Cd_4 clusters: (a) planar rhombus and (b) tetrahedron. The clusters represent in top view (top) and side view (bottom).

2. H_2 dissociation and H spillover

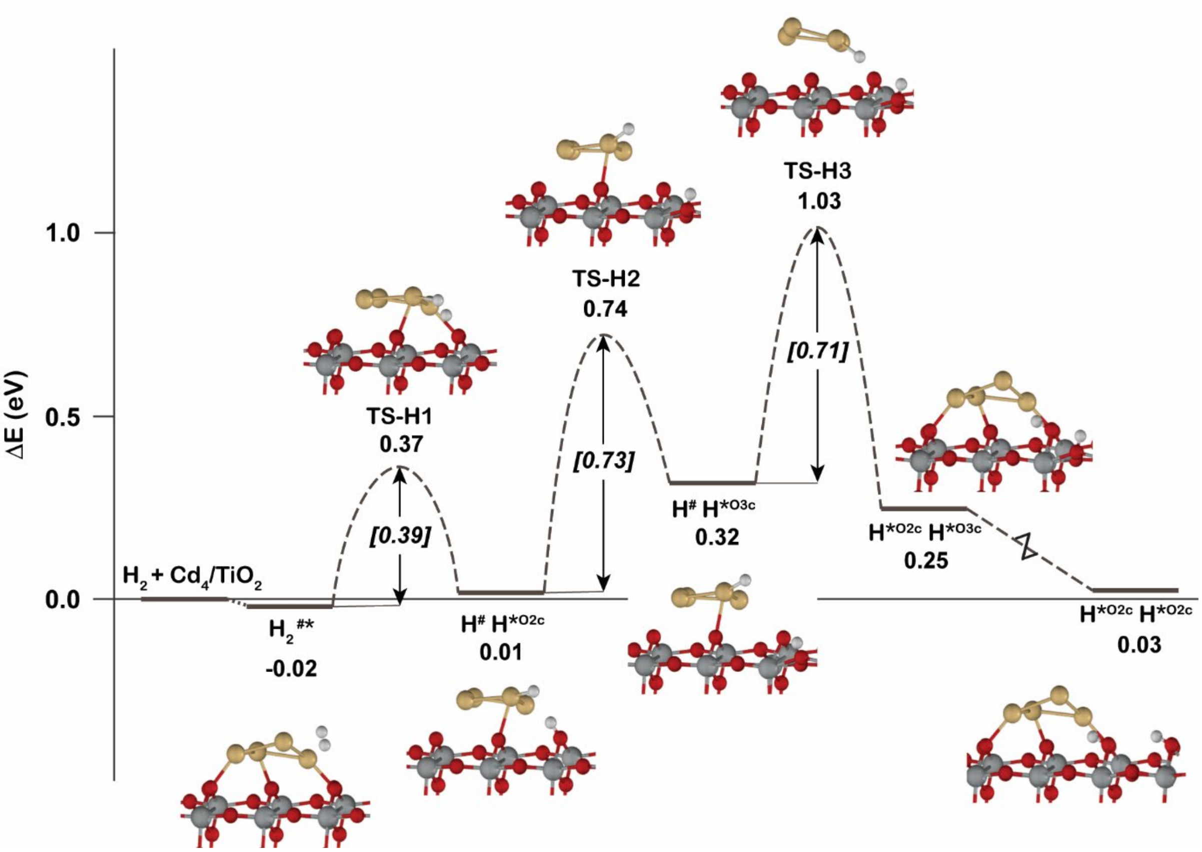

Figure S2. The reaction energy profiles for the H_2 dissociation followed by H migration and H spillover on Cd_4/TiO_2 catalyst

3. Reaction energy profiles

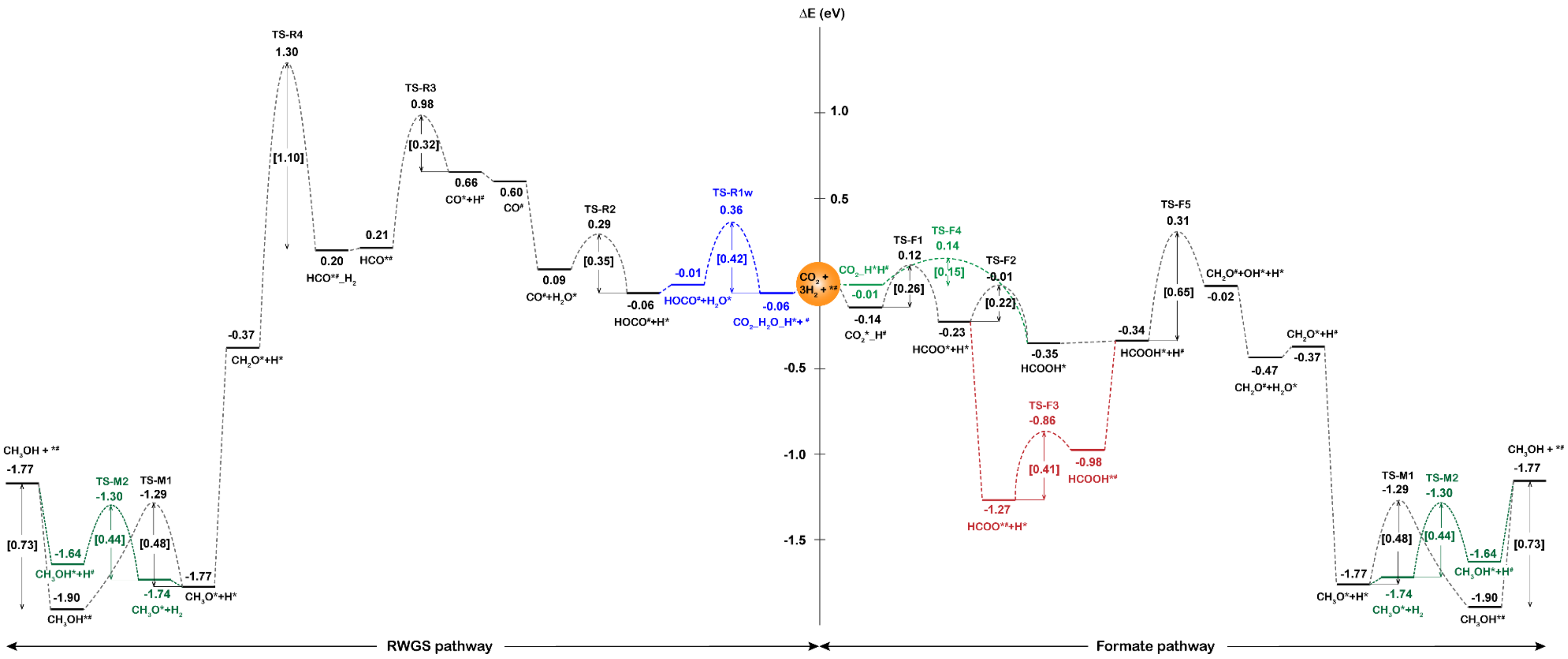

energy profiles for the CO_2 hydrogenation to CH_3OH on Cd_4/TiO_2 catalyst.
